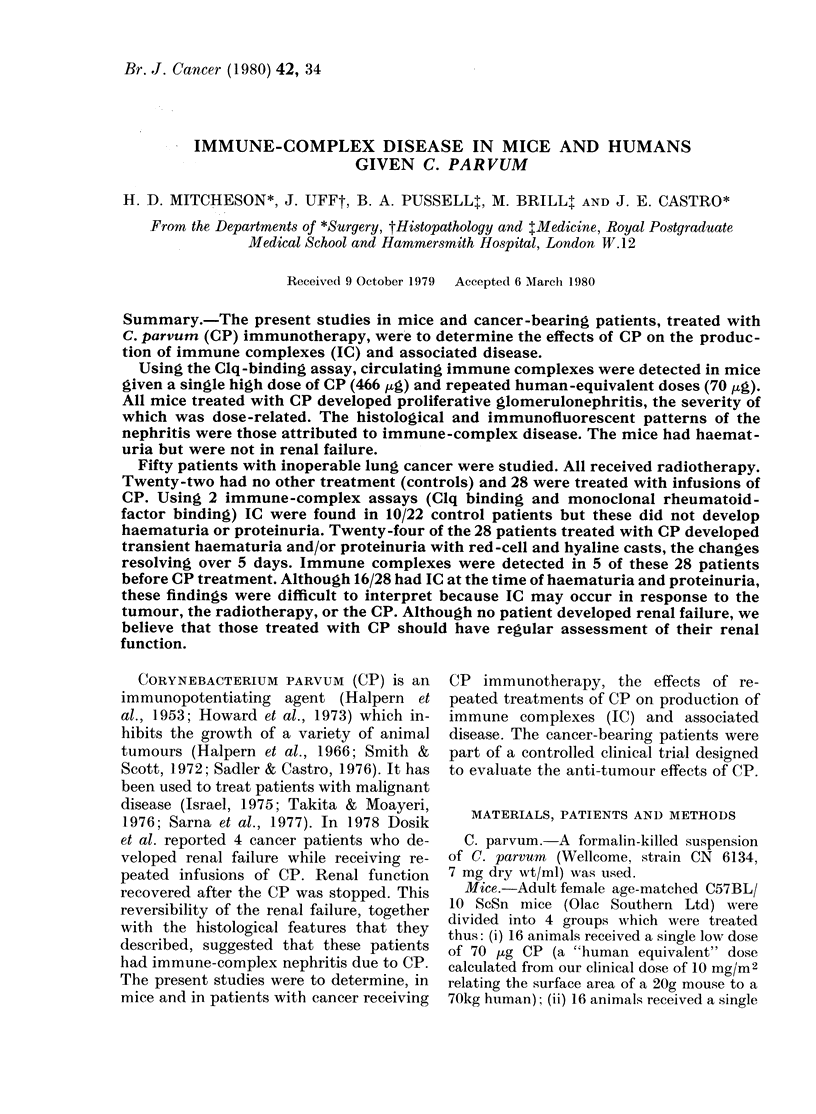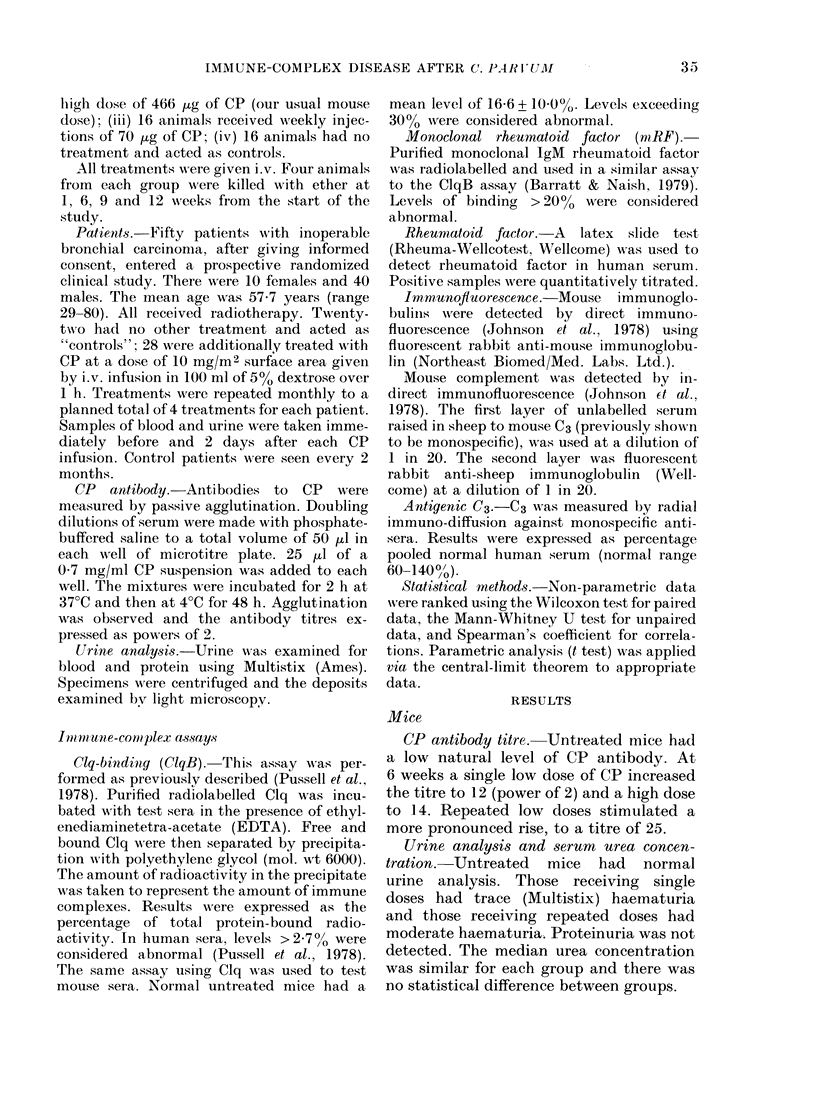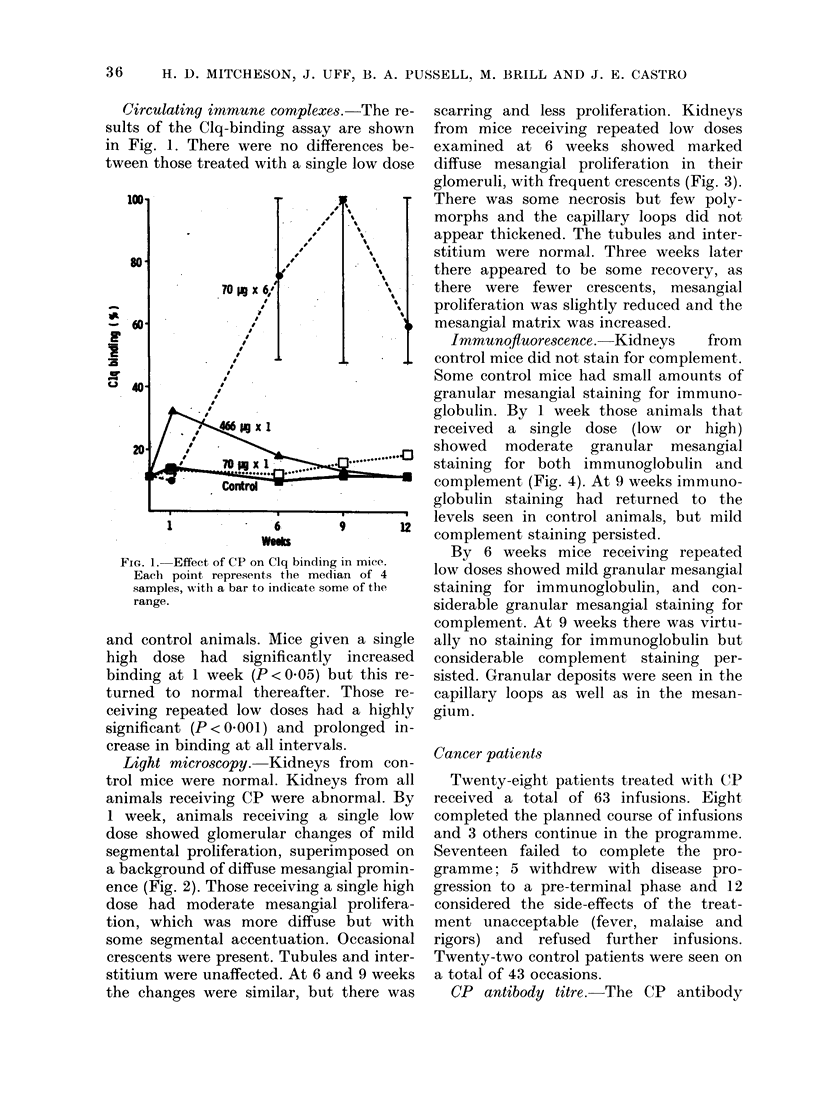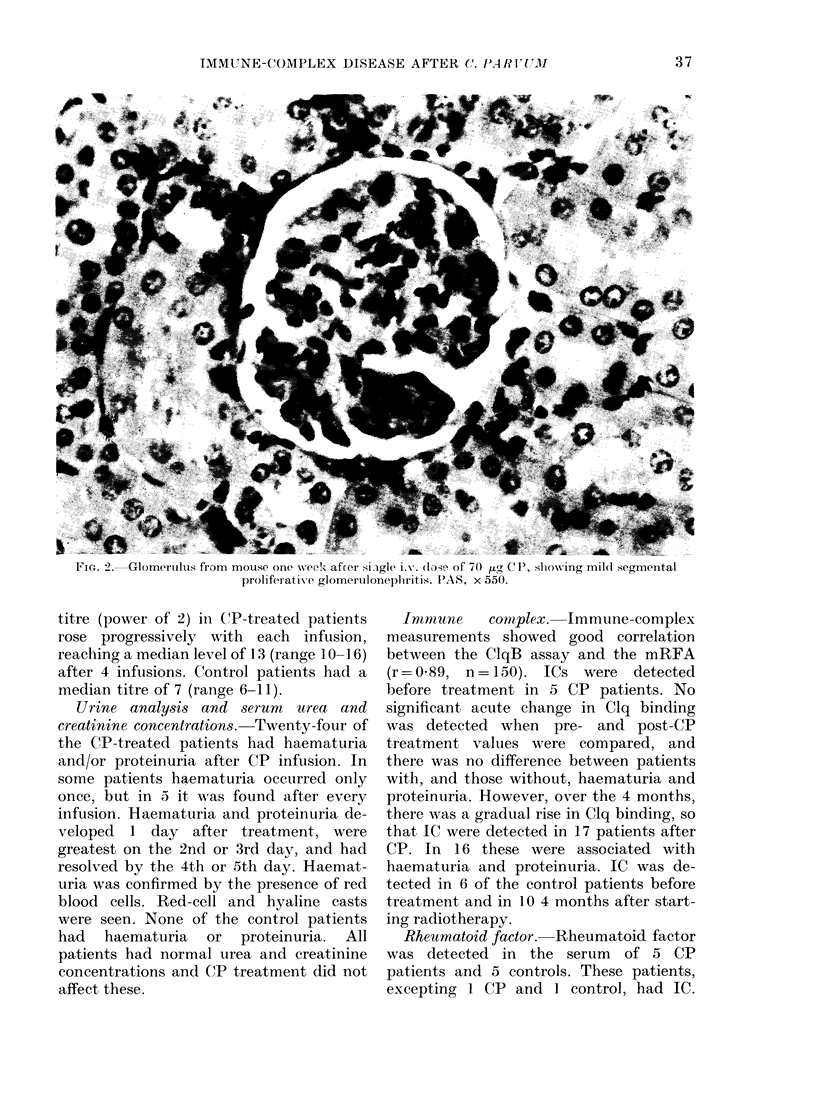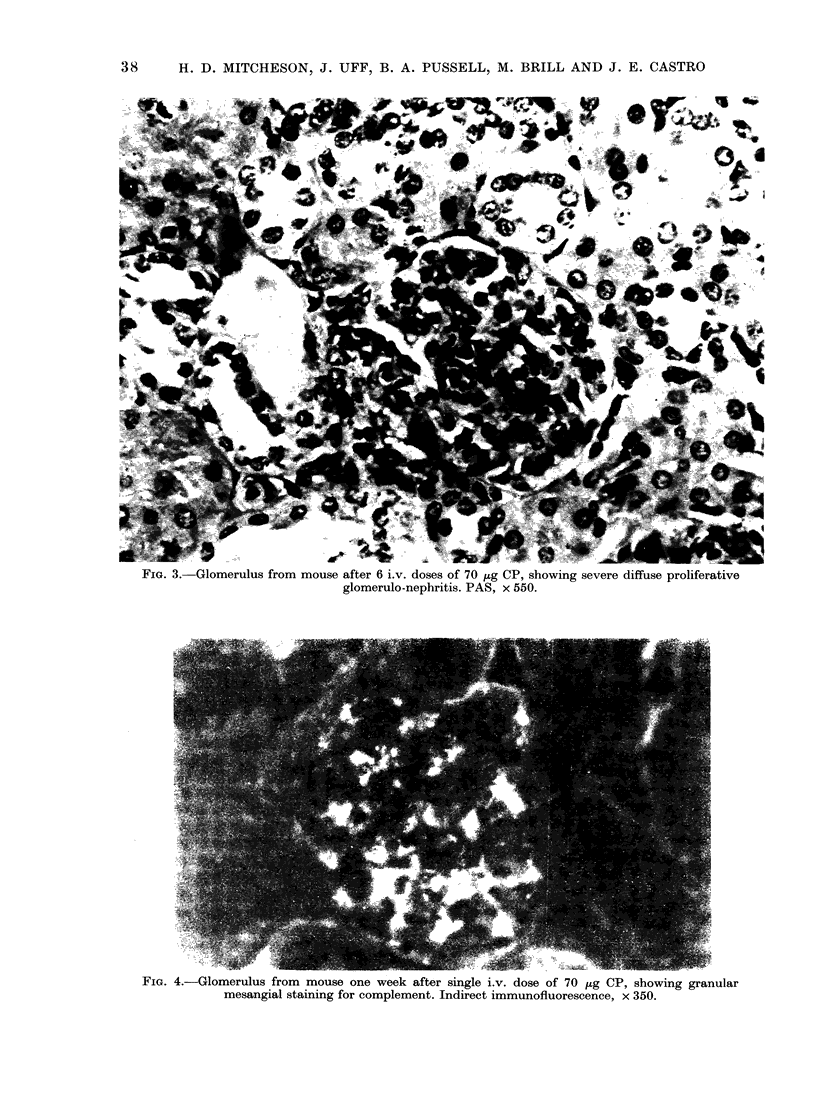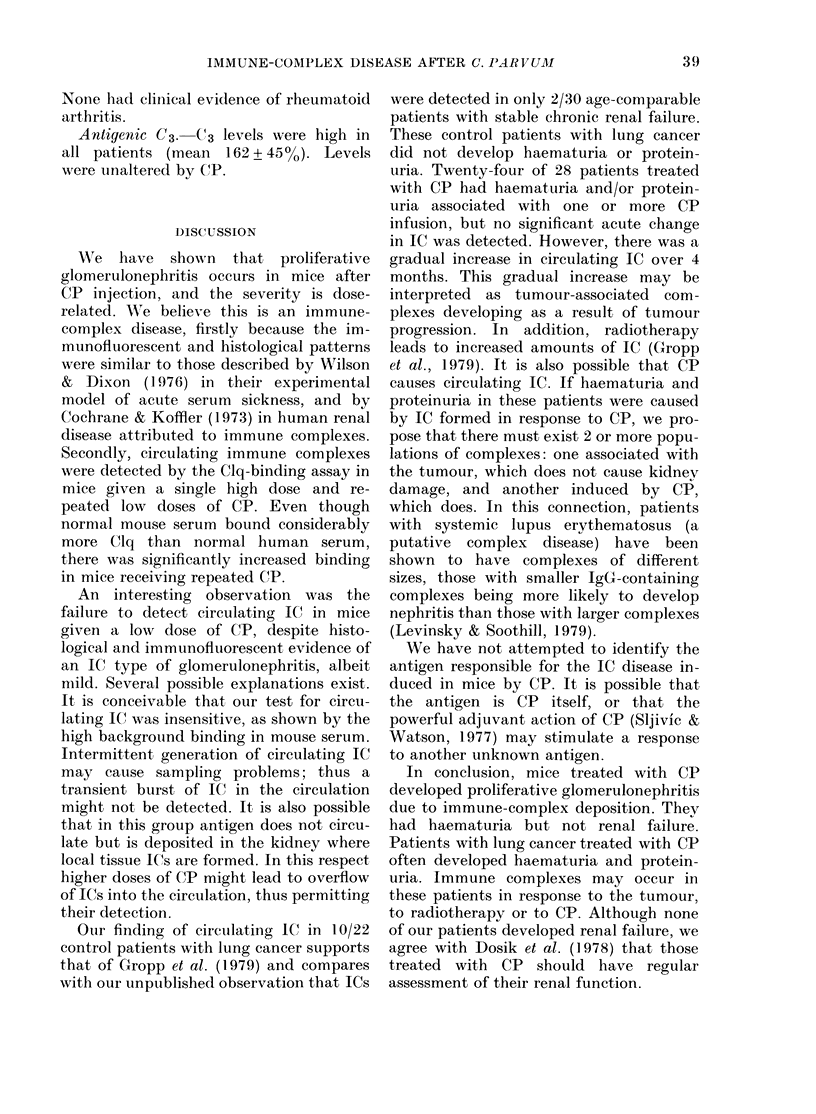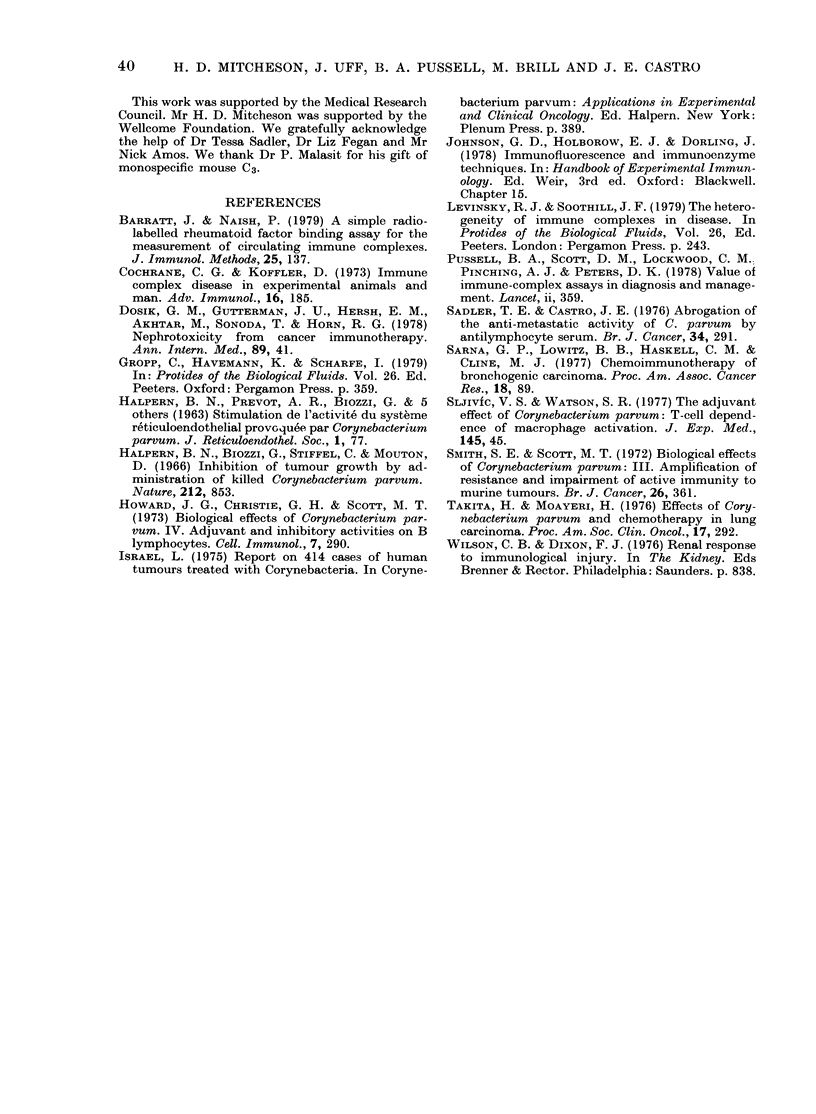# Immune-complex disease in mice and humans given C. parvum.

**DOI:** 10.1038/bjc.1980.200

**Published:** 1980-07

**Authors:** H. D. Mitcheson, J. Uff, B. A. Pussell, M. Brill, J. E. Castro

## Abstract

**Images:**


*Br. J. Cancer* (1980) **42**, 34

### IMMUNE-COMPLEX DISEASE IN MICE AND HUMANS GIVEN *C. PARVUM*

H. D. MITCHESON*, J. UFF†, B. A. PUSSELL‡, M. BRILL‡ AND J. E. CASTRO*

*From the Departments of *Surgery, †Histopathology and ‡Medicine, Royal Postgraduate Medical School and Hammersmith Hospital, London W.12*

Received 9 October 1979 Accepted 6 March 1980

**Summary.—The present studies in mice and cancer-bearing patients, treated with *C. parvum* (CP) immunotherapy, were to determine the effects of CP on the production of immune complexes (IC) and associated disease.**

**Using the Clq-binding assay, circulating immune complexes were detected in mice given a single high dose of CP (466 μg) and repeated human-equivalent doses (70 μg). All mice treated with CP developed proliferative glomerulonephritis, the severity of which was dose-related. The histological and immunofluorescent patterns of the nephritis were those attributed to immune-complex disease. The mice had haematuria but were not in renal failure.**

**Fifty patients with inoperable lung cancer were studied. All received radiotherapy. Twenty-two had no other treatment (controls) and 28 were treated with infusions of CP. Using 2 immune-complex assays (Clq binding and monoclonal rheumatoid-factor binding) IC were found in 10/22 control patients but these did not develop haematuria or proteinuria. Twenty-four of the 28 patients treated with CP developed transient haematuria and/or proteinuria with red-cell and hyaline casts, the changes resolving over 5 days. Immune complexes were detected in 5 of these 28 patients before CP treatment. Although 16/28 had IC at the time of haematuria and proteinuria, these findings were difficult to interpret because IC may occur in response to the tumour, the radiotherapy, or the CP. Although no patient developed renal failure, we believe that those treated with CP should have regular assessment of their renal function.**

CORYNEBACTERIUM PARVUM (CP) is an immunopotentiating agent (Halpern *et al.*, 1953; Howard *et al.*, 1973) which inhibits the growth of a variety of animal tumours (Halpern *et al.*, 1966; Smith & Scott, 1972; Sadler & Castro, 1976). It has been used to treat patients with malignant disease (Israel, 1975; Takita & Moayeri, 1976; Sarna *et al.*, 1977). In 1978 Dosik *et al.* reported 4 cancer patients who developed renal failure while receiving repeated infusions of CP. Renal function recovered after the CP was stopped. This reversibility of the renal failure, together with the histological features that they described, suggested that these patients had immune-complex nephritis due to CP. The present studies were to determine, in mice and in patients with cancer receiving CP immunotherapy, the effects of repeated treatments of CP on production of immune complexes (IC) and associated disease. The cancer-bearing patients were part of a controlled clinical trial designed to evaluate the anti-tumour effects of CP.

### MATERIALS, PATIENTS AND METHODS

C. parvum.—A formalin-killed suspension of *C. parvum* (Wellcome, strain CN 6134, 7 mg dry wt/ml) was used.

*Mice.*—Adult female age-matched C57BL/10 ScSn mice (Olac Southern Ltd) were divided into 4 groups which were treated thus: (i) 16 animals received a single low dose of 70 μg CP (a "human equivalent" dose calculated from our clinical dose of 10 mg/m$^2$ relating the surface area of a 20g mouse to a 70kg human); (ii) 16 animals received a single high dose of 466 μg of CP (our usual mouse dose); (iii) 16 animals received weekly injections of 70 μg of CP; (iv) 16 animals had no treatment and acted as controls.

All treatments were given i.v. Four animals from each group were killed with ether at 1, 6, 9 and 12 weeks from the start of the study.

*Patients.*—Fifty patients with inoperable bronchial carcinoma, after giving informed consent, entered a prospective randomized clinical study. There were 10 females and 40 males. The mean age was 57·7 years (range 29–80). All received radiotherapy. Twenty-two had no other treatment and acted as "controls"; 28 were additionally treated with CP at a dose of 10 mg/m$^2$ surface area given by i.v. infusion in 100 ml of 5% dextrose over 1 h. Treatments were repeated monthly to a planned total of 4 treatments for each patient. Samples of blood and urine were taken immediately before and 2 days after each CP infusion. Control patients were seen every 2 months.

*CP antibody.*—Antibodies to CP were measured by passive agglutination. Doubling dilutions of serum were made with phosphate-buffered saline to a total volume of 50 μl in each well of microtitre plate. 25 μl of a 0·7 mg/ml CP suspension was added to each well. The mixtures were incubated for 2 h at 37°C and then at 4°C for 48 h. Agglutination was observed and the antibody titres expressed as powers of 2.

*Urine analysis.*—Urine was examined for blood and protein using Multistix (Ames). Specimens were centrifuged and the deposits examined by light microscopy.

### *Immune-complex assays*

*Clq-binding* (*ClqB*).—This assay was performed as previously described (Pussell *et al.*, 1978). Purified radiolabelled Clq was incubated with test sera in the presence of ethylenediaminetetra-acetate (EDTA). Free and bound Clq were then separated by precipitation with polyethylene glycol (mol. wt 6000). The amount of radioactivity in the precipitate was taken to represent the amount of immune complexes. Results were expressed as the percentage of total protein-bound radioactivity. In human sera, levels >2·7% were considered abnormal (Pussell *et al.*, 1978). The same assay using Clq was used to test mouse sera. Normal untreated mice had a mean level of 16·6 ± 10·0%. Levels exceeding 30% were considered abnormal.

*Monoclonal rheumatoid factor* (*mRF*).—Purified monoclonal IgM rheumatoid factor was radiolabelled and used in a similar assay to the ClqB assay (Barratt & Naish, 1979). Levels of binding >20% were considered abnormal.

*Rheumatoid factor.*—A latex slide test (Rheuma-Wellcotest, Wellcome) was used to detect rheumatoid factor in human serum. Positive samples were quantitatively titrated.

*Immunofluorescence.*—Mouse immunoglobulins were detected by direct immunofluorescence (Johnson *et al.*, 1978) using fluorescent rabbit anti-mouse immunoglobulin (Northeast Biomed/Med. Labs. Ltd.).

Mouse complement was detected by indirect immunofluorescence (Johnson *et al.*, 1978). The first layer of unlabelled serum raised in sheep to mouse $C_3$ (previously shown to be monospecific), was used at a dilution of 1 in 20. The second layer was fluorescent rabbit anti-sheep immunoglobulin (Wellcome) at a dilution of 1 in 20.

*Antigenic $C_3$.*—$C_3$ was measured by radial immuno-diffusion against monospecific antisera. Results were expressed as percentage pooled normal human serum (normal range 60–140%).

*Statistical methods.*—Non-parametric data were ranked using the Wilcoxon test for paired data, the Mann-Whitney U test for unpaired data, and Spearman's coefficient for correlations. Parametric analysis (*t* test) was applied *via* the central-limit theorem to appropriate data.

### RESULTS

### *Mice*

*CP antibody titre.*—Untreated mice had a low natural level of CP antibody. At 6 weeks a single low dose of CP increased the titre to 12 (power of 2) and a high dose to 14. Repeated low doses stimulated a more pronounced rise, to a titre of 25.

*Urine analysis and serum urea concentration.*—Untreated mice had normal urine analysis. Those receiving single doses had trace (Multistix) haematuria and those receiving repeated doses had moderate haematuria. Proteinuria was not detected. The median urea concentration was similar for each group and there was no statistical difference between groups.

*Circulating immune complexes.*—The results of the Clq-binding assay are shown in Fig. 1. There were no differences between those treated with a single low dose and control animals. Mice given a single high dose had significantly increased binding at 1 week ($P < 0{\cdot}05$) but this returned to normal thereafter. Those receiving repeated low doses had a highly significant ($P < 0{\cdot}001$) and prolonged increase in binding at all intervals.

FIG. 1.—Effect of CP on Clq binding in mice. Each point represents the median of 4 samples, with a bar to indicate some of the range.

*Light microscopy.*—Kidneys from control mice were normal. Kidneys from all animals receiving CP were abnormal. By 1 week, animals receiving a single low dose showed glomerular changes of mild segmental proliferation, superimposed on a background of diffuse mesangial prominence (Fig. 2). Those receiving a single high dose had moderate mesangial proliferation, which was more diffuse but with some segmental accentuation. Occasional crescents were present. Tubules and interstitium were unaffected. At 6 and 9 weeks the changes were similar, but there was scarring and less proliferation. Kidneys from mice receiving repeated low doses examined at 6 weeks showed marked diffuse mesangial proliferation in their glomeruli, with frequent crescents (Fig. 3). There was some necrosis but few polymorphs and the capillary loops did not appear thickened. The tubules and interstitium were normal. Three weeks later there appeared to be some recovery, as there were fewer crescents, mesangial proliferation was slightly reduced and the mesangial matrix was increased.

*Immunofluorescence.*—Kidneys from control mice did not stain for complement. Some control mice had small amounts of granular mesangial staining for immunoglobulin. By 1 week those animals that received a single dose (low or high) showed moderate granular mesangial staining for both immunoglobulin and complement (Fig. 4). At 9 weeks immunoglobulin staining had returned to the levels seen in control animals, but mild complement staining persisted.

By 6 weeks mice receiving repeated low doses showed mild granular mesangial staining for immunoglobulin, and considerable granular mesangial staining for complement. At 9 weeks there was virtually no staining for immunoglobulin but considerable complement staining persisted. Granular deposits were seen in the capillary loops as well as in the mesangium.

*Cancer patients*

Twenty-eight patients treated with CP received a total of 63 infusions. Eight completed the planned course of infusions and 3 others continue in the programme. Seventeen failed to complete the programme; 5 withdrew with disease progression to a pre-terminal phase and 12 considered the side-effects of the treatment unacceptable (fever, malaise and rigors) and refused further infusions. Twenty-two control patients were seen on a total of 43 occasions.

*CP antibody titre.*—The CP antibody

FIG. 2.—Glomerulus from mouse one week after single i.v. dose of 70 $\mu g$ CP, showing mild segmental proliferative glomerulonephritis. PAS, × 550.

titre (power of 2) in CP-treated patients rose progressively with each infusion, reaching a median level of 13 (range 10–16) after 4 infusions. Control patients had a median titre of 7 (range 6–11).

*Urine analysis and serum urea and creatinine concentrations.*—Twenty-four of the CP-treated patients had haematuria and/or proteinuria after CP infusion. In some patients haematuria occurred only once, but in 5 it was found after every infusion. Haematuria and proteinuria developed 1 day after treatment, were greatest on the 2nd or 3rd day, and had resolved by the 4th or 5th day. Haematuria was confirmed by the presence of red blood cells. Red-cell and hyaline casts were seen. None of the control patients had haematuria or proteinuria. All patients had normal urea and creatinine concentrations and CP treatment did not affect these.

*Immune complex.*—Immune-complex measurements showed good correlation between the ClqB assay and the mRFA ($r = 0{\cdot}89$, $n = 150$). ICs were detected before treatment in 5 CP patients. No significant acute change in Clq binding was detected when pre- and post-CP treatment values were compared, and there was no difference between patients with, and those without, haematuria and proteinuria. However, over the 4 months, there was a gradual rise in Clq binding, so that IC were detected in 17 patients after CP. In 16 these were associated with haematuria and proteinuria. IC was detected in 6 of the control patients before treatment and in 10 4 months after starting radiotherapy.

*Rheumatoid factor.*—Rheumatoid factor was detected in the serum of 5 CP patients and 5 controls. These patients, excepting 1 CP and 1 control, had IC.

FIG. 3.—Glomerulus from mouse after 6 i.v. doses of 70 $\mu$g CP, showing severe diffuse proliferative glomerulo-nephritis. PAS, $\times$ 550.

FIG. 4.—Glomerulus from mouse one week after single i.v. dose of 70 $\mu$g CP, showing granular mesangial staining for complement. Indirect immunofluorescence, $\times$ 350.

None had clinical evidence of rheumatoid arthritis.

*Antigenic* $C_3$.—$C_3$ levels were high in all patients (mean $162 \pm 45\%$). Levels were unaltered by CP.

### DISCUSSION

We have shown that proliferative glomerulonephritis occurs in mice after CP injection, and the severity is dose-related. We believe this is an immune-complex disease, firstly because the immunofluorescent and histological patterns were similar to those described by Wilson & Dixon (1976) in their experimental model of acute serum sickness, and by Cochrane & Koffler (1973) in human renal disease attributed to immune complexes. Secondly, circulating immune complexes were detected by the Clq-binding assay in mice given a single high dose and repeated low doses of CP. Even though normal mouse serum bound considerably more Clq than normal human serum, there was significantly increased binding in mice receiving repeated CP.

An interesting observation was the failure to detect circulating IC in mice given a low dose of CP, despite histological and immunofluorescent evidence of an IC type of glomerulonephritis, albeit mild. Several possible explanations exist. It is conceivable that our test for circulating IC was insensitive, as shown by the high background binding in mouse serum. Intermittent generation of circulating IC may cause sampling problems; thus a transient burst of IC in the circulation might not be detected. It is also possible that in this group antigen does not circulate but is deposited in the kidney where local tissue ICs are formed. In this respect higher doses of CP might lead to overflow of ICs into the circulation, thus permitting their detection.

Our finding of circulating IC in 10/22 control patients with lung cancer supports that of Gropp *et al.* (1979) and compares with our unpublished observation that ICs were detected in only 2/30 age-comparable patients with stable chronic renal failure. These control patients with lung cancer did not develop haematuria or proteinuria. Twenty-four of 28 patients treated with CP had haematuria and/or proteinuria associated with one or more CP infusion, but no significant acute change in IC was detected. However, there was a gradual increase in circulating IC over 4 months. This gradual increase may be interpreted as tumour-associated complexes developing as a result of tumour progression. In addition, radiotherapy leads to increased amounts of IC (Gropp *et al.*, 1979). It is also possible that CP causes circulating IC. If haematuria and proteinuria in these patients were caused by IC formed in response to CP, we propose that there must exist 2 or more populations of complexes: one associated with the tumour, which does not cause kidney damage, and another induced by CP, which does. In this connection, patients with systemic lupus erythematosus (a putative complex disease) have been shown to have complexes of different sizes, those with smaller IgG-containing complexes being more likely to develop nephritis than those with larger complexes (Levinsky & Soothill, 1979).

We have not attempted to identify the antigen responsible for the IC disease induced in mice by CP. It is possible that the antigen is CP itself, or that the powerful adjuvant action of CP (Sljivíc & Watson, 1977) may stimulate a response to another unknown antigen.

In conclusion, mice treated with CP developed proliferative glomerulonephritis due to immune-complex deposition. They had haematuria but not renal failure. Patients with lung cancer treated with CP often developed haematuria and proteinuria. Immune complexes may occur in these patients in response to the tumour, to radiotherapy or to CP. Although none of our patients developed renal failure, we agree with Dosik *et al.* (1978) that those treated with CP should have regular assessment of their renal function.

This work was supported by the Medical Research Council. Mr H. D. Mitcheson was supported by the Wellcome Foundation. We gratefully acknowledge the help of Dr Tessa Sadler, Dr Liz Fegan and Mr Nick Amos. We thank Dr P. Malasit for his gift of monospecific mouse $C_3$.

### REFERENCES

BARRATT, J. & NAISH, P. (1979) A simple radiolabelled rheumatoid factor binding assay for the measurement of circulating immune complexes. *J. Immunol. Methods*, **25**, 137.

COCHRANE, C. G. & KOFFLER, D. (1973) Immune complex disease in experimental animals and man. *Adv. Immunol.*, **16**, 185.

DOSIK, G. M., GUTTERMAN, J. U., HERSH, E. M., AKHTAR, M., SONODA, T. & HORN, R. G. (1978) Nephrotoxicity from cancer immunotherapy. *Ann. Intern. Med.*, **89**, 41.

GROPP, C., HAVEMANN, K. & SCHARFE, I. (1979) In: *Protides of the Biological Fluids*. Vol. 26. Ed. Peeters. Oxford: Pergamon Press. p. 359.

HALPERN, B. N., PREVOT, A. R., BIOZZI, G. & 5 others (1963) Stimulation de l'activité du système réticuloendothelial provoquée par *Corynebacterium parvum*. *J. Reticuloendothel. Soc.*, **1**, 77.

HALPERN, B. N., BIOZZI, G., STIFFEL, C. & MOUTON, D. (1966) Inhibition of tumour growth by administration of killed *Corynebacterium parvum*. *Nature*, **212**, 853.

HOWARD, J. G., CHRISTIE, G. H. & SCOTT, M. T. (1973) Biological effects of *Corynebacterium parvum*. IV. Adjuvant and inhibitory activities on B lymphocytes. *Cell. Immunol.*, **7**, 290.

ISRAEL, L. (1975) Report on 414 cases of human tumours treated with Corynebacteria. In Corynebacterium parvum: *Applications in Experimental and Clinical Oncology*. Ed. Halpern. New York: Plenum Press. p. 389.

JOHNSON, G. D., HOLBOROW, E. J. & DORLING, J. (1978) Immunofluorescence and immunoenzyme techniques. In: *Handbook of Experimental Immunology*. Ed. Weir, 3rd ed. Oxford: Blackwell. Chapter 15.

LEVINSKY, R. J. & SOOTHILL, J. F. (1979) The heterogeneity of immune complexes in disease. In *Protides of the Biological Fluids*, Vol. 26, Ed. Peeters. London: Pergamon Press. p. 243.

PUSSELL, B. A., SCOTT, D. M., LOCKWOOD, C. M., PINCHING, A. J. & PETERS, D. K. (1978) Value of immune-complex assays in diagnosis and management. *Lancet*, ii, 359.

SADLER, T. E. & CASTRO, J. E. (1976) Abrogation of the anti-metastatic activity of *C. parvum* by antilymphocyte serum. *Br. J. Cancer*, **34**, 291.

SARNA, G. P., LOWITZ, B. B., HASKELL, C. M. & CLINE, M. J. (1977) Chemoimmunotherapy of bronchogenic carcinoma. *Proc. Am. Assoc. Cancer Res.*, **18**, 89.

SLJIVÍC, V. S. & WATSON, S. R. (1977) The adjuvant effect of *Corynebacterium parvum*: T-cell dependence of macrophage activation. *J. Exp. Med.*, **145**, 45.

SMITH, S. E. & SCOTT, M. T. (1972) Biological effects of *Corynebacterium parvum*: III. Amplification of resistance and impairment of active immunity to murine tumours. *Br. J. Cancer*, **26**, 361.

TAKITA, H. & MOAYERI, H. (1976) Effects of *Corynebacterium parvum* and chemotherapy in lung carcinoma. *Proc. Am. Soc. Clin. Oncol.*, **17**, 292.

WILSON, C. B. & DIXON, F. J. (1976) Renal response to immunological injury. In *The Kidney*. Eds Brenner & Rector. Philadelphia: Saunders. p. 838.